# Unraveling the plant microbiome: looking back and future perspectives

**DOI:** 10.3389/fmicb.2014.00148

**Published:** 2014-06-04

**Authors:** Gabriele Berg, Martin Grube, Michael Schloter, Kornelia Smalla

**Affiliations:** ^1^Austrian Centre of Industrial BiotechnologyGraz, Austria; ^2^Institute of Environmental Biotechnology, Graz University of TechnologyGraz, Austria; ^3^Institute of Plant Sciences, University of GrazGraz, Austria; ^4^Environmental Genomics, Helmholtz Zentrum MünchenOberschleissheim, Germany; ^5^Julius Kühn-Institute (JKI), Institute for Epidemiology and Pathogen Diagnostics, Federal Research Centre for Cultivated PlantsBraunschweig, Germany

**Keywords:** meta-organisms, plant microbiome, plant-microbe interaction, biocontrol, stress protection, plant growth promotion

## Abstract

Most eukaryotes develop close interactions with microorganisms that are essential for their performance and survival. Thus, eukaryotes and prokaryotes in nature can be considered as meta-organisms or holobionts. Consequently, microorganisms that colonize different plant compartments contain the plant’s second genome. In this respect, many studies in the last decades have shown that plant-microbe interactions are not only crucial for better understanding plant growth and health, but also for sustainable crop production in a changing world. This mini-review acting as editorial presents retrospectives and future perspectives for plant microbiome studies as well as information gaps in this emerging research field. In addition, the contribution of this research topic to the solution of various issues is discussed.

## INTRODUCTION AND RETROSPECT ON THE STUDY OF PLANT-ASSOCIATED MICROORGANISMS

Many studies on plant-associated microorganisms reflect the enormous interest in this topic and the full effect of ongoing research (Bulgarelli et al., 2013). Due to the importance of the soil habitat of plants, the majority of research focuses on the rhizosphere, even though microorganisms are also able to readily colonize most plant compartments. Several recent reviews addressed particular aspects of plant microbiome research. The current knowledge of rhizosphere inhabitants, their function, and their promising biotechnological potential was summarized by Hirsch and Mauchline (2012), Bakker et al. (2013), Mendes et al. (2013). Berendsen et al. (2012) reviewed more specifically the plant microbiome and plant health relationship, while Berg et al. (2005a) focused on the occurrence of potential human pathogenic bacteria in the rhizosphere. The important question about the factors contributing to selective enrichment of microorganisms from the soil into the rhizosphere was addressed by Bais et al. (2006), Doornbos et al. (2012). It now appears that in addition to carbohydrates and even amino acids which act as general chemical determinants in the rhizosphere (Moe, 2013), secondary metabolites such as plant-specific flavonoids were identified as key drivers in the development of plant-specific microbial communities in the rhizosphere (Weston and Mathesius, 2013).

While the well-studied rhizosphere presents the soil-plant interface, the phyllosphere forms the air-plant interface. This microhabitat is also of special interest due to its large and exposed surface area and its connection to the air microbiome, especially air-borne pathogens (rev. in Lindow and Brandl, 2003; Vorholt, 2012; Rastogi et al., 2013). However, in addition to the well-studied rhizo- and phyllospheres, each plant can be divided into more microenvironments, e.g., the endorhiza (root), the anthosphere (flower), the spermosphere (seeds), and the carposphere (fruit). Moreover, we generally differentiate between the endosphere (inner tissues) and ectosphere (outer surfaces; Ryan et al., 2008). All these microenvironments provide specific biotic and abiotic conditions for microbial life, which also have a correspondingly specific function for the host. The potential of these findings and the use of plant growth-promoting bacteria and biocontrol agents for the development of sustainable forms of agricultural management were discussed by Leveau (2007), Köberl et al. (2012), Berg et al. (2013).

The first section of this editorial focuses on several historical milestones in plant microbiome research. Despite the enormous progress already made, many challenges still exist. We address some information gaps in the second section of this editorial, and conclude with an overview of the present contributions. The papers in this special issue focus mainly on the bacterial dimension of the plant-associated microbiome, and we will show how they complement and extend the current research and how they will spur further questions.

## THE RHIZOSPHERE WAS DEFINED MORE THAN A CENTURY AGO

**FIGURE 1:**
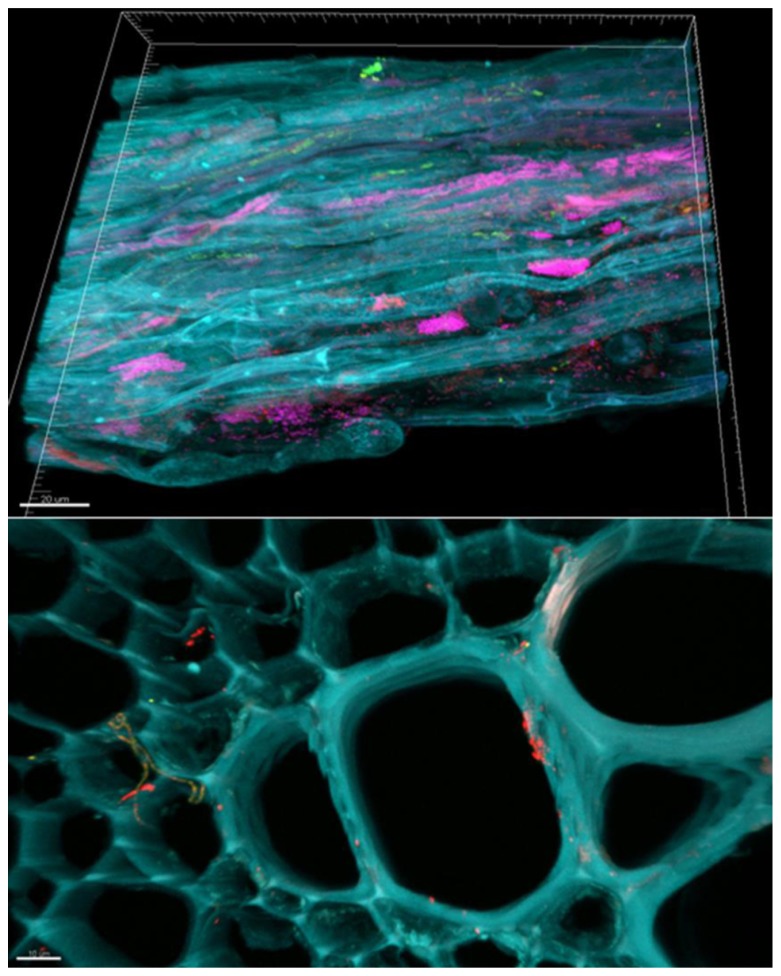
**Bacterial micro-colonies in the rhizosphere and endosphere visualized by fluorescence *in situ* hybridization (FISH) and confocal laser scanning microscopy(CLSM).** The rhizosphere microbiome of lettuce is dominated by Betaproteobacteria (purple) forming dense colonies on the root surface. The endosphere is shown as section of the main root of a lettuce plant. While unspecifically labeled bacteria are in red, Alphaproteobacteria (orange-green) are to be seen as colonies as well as filamentous forms.

Hiltner (1904) defined the “rhizosphere” as root-surrounding soil influenced by root exudates (Hartmann et al., 2008). In addition, he was the first to suggest the importance of microbial root inhabitants for plant growth and health. The rhizosphere is of central importance not only for plant nutrition, health, and quality. Today we are aware of microorganism-driven carbon sequestration in this ecological niche, which has an important role in ecosystem functioning and nutrient cycling in terrestrial ecosystems. In contrast to the other microenvironment of plants, the rhizosphere is characterized by high microbial abundances (Berg et al., 2005b) and activities (Herron et al., 2013). Due to the densely colonized surface and surrounding soil (**Figure 1**), the rhizosphere was suggested as a protection shield against soil-borne pathogens (Weller et al., 2002).

## THE ENDOSPHERE IS A HABITAT FOR INTIMATE INTERACTIONS

Although endophytes were ignored or considered contaminants for a long time, many endophytic inhabitants of plants are now often recognized as symbionts with a unique and intimate interaction with the plant (Ryan et al., 2008; Reinhold-Hurek and Hurek, 2011; Mitter et al., 2013). In these and other more recent studies, evidence of the occurrence of endophytes was assessed by cultivation-independent analyses, and via fluorescence *in situ* hybridization-confocal laser scanning microscopy (FISH-CLSM; an example for endophytes in the lettuce endorhiza is shown in **Figure 1**). After the first definition by De Bary (1866) as “any organism occurring within plant tissues,” various researchers have defined endophytes in different ways, which were usually related to their own research context and perspective (Wilson, 1995; Hallmann et al., 1997; Bacon and White, 2000).

## THE FUNCTIONS OF THE PLANT MICROBIOME ARE ESSENTIAL FOR THE HOST

Plant-associated microorganisms can help plants to suppress diseases, to stimulate growth, to occupy space that would otherwise be available to pathogens, to promote stress resistance, and influence crop yield and quality by nutrient mobilization and transport (Lugtenberg and Kamilova, 2009; Yang et al., 2009). Therefore, the plant microbiome is one of the key determinants of plant health and productivity. Additional essential roles of the plant microbiome for phenotypic and epigenetic plasticity as well as the evolution of plants were suggested by Partida-Martínez and Heil (2011).

## SPECIFIC ENRICHMENT OF MICROORGANISMS IN PLANT-ASSOCIATED COMMUNITIES EXISTS

So far, research on the specificity of plant-associated microbiomes focused on the rhizosphere, while only few other compartments have been studied in this respect (Vorholt, 2012). Although plant specific microbiomes in the rhizosphere have already been postulated via cultivation-based approaches (Germida and Siciliano, 2001), molecular fingerprints provided the first clear evidence for plant-dependent microbial community compositions (Smalla et al., 2001). Differences in plant root exudates play an important role as both chemo-attractants as well as repellents (Badri and Vivanco, 2009), to which bacteria are especially responsive (Costa et al., 2006, 2007; DeAngelis et al., 2009). In addition, plant defense signaling plays a role in this process as well (Doornbos et al., 2012). Haichar et al. (2008) used a stable isotope probing (SIP) approach to show that plant host habitat and root exudates shape the soil bacterial community structure. Thus, the plant is clearly able to select microorganisms for rhizosphere colonization primarily from the large pool living in the surrounding soil. Lundberg et al. (2012), Bulgarelli et al. (2012) revealed that only a subset of the bacterial community in the soil is present around the plant roots of *Arabidopsis thaliana* through amplicon sequencing of 16S rRNA gene fragments. Furthermore, the use of catalyzed reporter deposition and *in situ* hybridization or FISH was used to confirm the co-localization and dynamics of dominant taxa determined by 454 pyrosequencing (Bulgarelli et al., 2012; Lundberg et al., 2012; Ofek et al., 2012). While the use of FISH and catalyzed reported deposition-fluorescence in situ hybridization (CARD-FISH) helped to unravel the spatial distribution of dominant indigenous bacterial communities, the use of marker and reporter genes was employed in several studies to localize inoculated potential biocontrol strains and to measure distributions of nutrients, metals, and organic exudates along the roots on a microscale (Sørensen et al., 2009).

However, the plant (species, cultivar, age, health, and developmental stage) is not the only factor that influences microbial communities in the rhizosphere: a multitude of abiotic factors modulate the structural and functional diversity of the rhizosphere microbiome, including soil properties, nutrient status, and climatic conditions (rev. in Berg and Smalla, 2009). Moreover, large-scale agricultural management such as manure application has a clear impact on the microbiome composition (Jechalke et al., 2014).

## THE ORIGIN OF PLANT-ASSOCIATED BACTERIA IS DIFFERENT

Plants are in constant contact with diverse microorganisms originating either through soil, wind, and air, or water via the water cycle. After initial exposure, some of these microorganisms are able to colonize the plant and survive (Rastogi et al., 2012). In some cases, microorganisms can even be transferred vertically from the parent plants to their progeny. Endophytes present in plant seeds may subsequently colonize the roots and the rhizosphere (Johnston-Monje and Raizada, 2011; Links et al., 2014). In addition, generative organs such as anther pockets, producing pollen (Fürnkranz et al., 2012), and moss sporophytes (Bragina et al., 2012) share a microbiome containing beneficials with their host plant.

## *Pseudomonas* AND *Bacillus* ARE MODEL PLANT-ASSOCIATED BACTERIA

Although we now know that plant-associated bacteria are phylogenetically diverse, *Pseudomonas* and *Bacillus* have been studied as models for beneficial plant-microbe interaction (Emmert and Handelsman, 1999; Weller et al., 2002; Raaijmakers et al., 2010) for a long time. Interestingly, the importance of both genera on plants has been corroborated in many metagenomic studies. While *Pseudomonas* is abundant under humid conditions (Mendes et al., 2012), *Bacillus* dominates plant microbiomes under arid conditions such as in Egypt where *Pseudomonas* cannot survive (Köberl et al., 2011). The more detailed information obtained for *Pseudomona*s–plant interactions now help in understanding the bigger picture of *Pseudomonas* genome–plant interaction in its entirety as shown in the excellent review by Loper et al. (2012).

Antibiotic production by plant-associated microorganisms, with the rhizosphere and endosphere as a “hot spot” for potential producers, is a further aspect of research, for which both model organisms again play an important role. *Pseudomonas* is known for its versatile antibiotic production, which has also been shown *in situ* in the rhizosphere (Bonsall et al., 1997). Yet, a lot has still to be learned about the diffusion and action of small molecule antibiotics. Antibiotics are not only acting in solutes, some bioactive compounds act as volatiles, both in antibiosis against pathogens as well as in communication with plants (Ryu et al., 2003). According to recent reports, antibiotics and lipopeptides of bacteria are regulators and support biofilm formation, signaling, motility, and acquisition of micronutrients at sub-inhibitory concentrations (Raaijmakers et al., 2010; Raaijmakers and Mazzola, 2012). An interesting regulatory network was also detected for redox-active antibiotics such as phenazine, which is also involved in the reduction of Fe^3^^+^(Raaijmakers and Mazzola, 2012). This high number of antibiotic producers associated with plants may have driven the evolutions of resistance genes as well (Allen et al., 2010).

Several studies, which focused primarily on *Pseudomonas* demonstrated bacterial intra- and interspecies communication in the plant-soil interface plant-microbe interaction via quorum sensing molecules such as *N*-acyl homoserine lactones (*N*-AHLs), or antibiotics at sub-inhibitory concentration (Steidle et al., 2001; DeAngelis et al., 2009; Hartmann and Schikora, 2012; Raaijmakers and Mazzola, 2012). Bacterial AHLs were demonstrated to change the plant transcriptome, modify root growth, and induce systemic resistance to phytopathogens (von Rad et al., 2008; Hartmann and Schikora, 2012; Raaijmakers and Mazzola, 2012); yet substantial differences were observed in the uptake, transport, and degradation of various AHLs for different plants (Götz et al., 2007).

## HORIZONTAL GENE TRANSFER CONTRIBUTES TO PLASTICITY AND EVOLUTION OF PLANT-ASSOCIATED BACTERIA

Owing to the availability of various nutrients and surfaces, the plant-soil interface is also considered a hot spot for horizontal gene transfer processes via plasmids (Heuer and Smalla, 2012). The recent progress in microscopy tools has been extremely helpful in gaining further insight into the spatial distribution and dynamics of the plant-soil interface. Plant species-dependent differences were observed for the conjugation of a *gfp*-tagged IncP-1ε plasmid that did not express the *gfp* in its original host due to the presence of a *lac*-repressor (Mølbak et al., 2007). Through *in situ* visualization, these authors could demonstrate that both exudation patterns and root growth rates determined plasmid transfer in the pea and barley rhizospheres.

## FUTURE PERSPECTIVES AND INFORMATION GAPS

Although the plant microbiome is recognized as an immense treasure trove of microbial diversity, numerous important crop species and their natural relatives have not yet been studied for their associated bacterial communities. With an approximate number of 500,000 plant species a lot of work lays ahead of plant microbiome research to explore new aspects about phylogenetic diversity of plant-associated microorganisms in the future. This might be particularly interesting with plants from extreme natural ecosystems or with unique life styles (carnivores, parasites, etc.).

Despite this enormous progress in the description of the plant microbiome, more fundamental and practical studies to address the processes leading to community assembly and function in and on plants are needed. Metagenomic analysis and comparison of plant-associated communities will lead to novel phylogenetic and functional insight. The first metagenomes, -proteomes, and -transcriptomes are currently published (Delmotte et al., 2009; Knief et al., 2012). An interesting example for a novel function is the detection of potential coexistence of microbial and plant photosynthesis on *Tamarix* leaves (Atamna-Ismaeel et al., 2012). Functional analysis will demonstrate whether the plants are able to benefit from the presence of certain microorganisms. In this context it should also be kept in mind that activation patterns and induction pathways can differ between ecotypes and strains.

Amplicon sequencing of 16S rRNA gene fragments provided valuable insight into the dominant colonizers, but too much emphasis on this locus may underdiagnose the potential biological variation. For example, biological functions provided from the mobilome (Eltlbany et al., 2012) do not correspond with 16S rRNA gene data. In addition, ribosomal gene amplicon quantities can depend on extraction methods, primer efficiency (Pinto and Raskin, 2012), and their copy-number variation (Kembel et al., 2012).

Although with the following articles in this special issue focus was given to the bacterial aspect of plant microbiomes we predict a future integration with fungal–bacterial interactions, specifically in the context of mycorrhiza (Bonfante and Anca, 2009; Song et al., 2010).

Plant microbiome discoveries could fuel advances in sustainable agriculture (Berg, 2009; Lugtenberg and Kamilova, 2009), such as the development of microbial inoculants as biofertilizers, biocontrol, or stress protection products (Berg, 2009; Berg et al., 2013).

In the future, the plant microbiome will have a greater importance for plant breeding and plant biotechnology. Until now, primarily plant pathogens were considered in these approaches. However, we suggest that the beneficial aspect of the entire microbiome should also be integrated as a biomarker.

A better understanding of the whole plant microbiome might be important to prevent outbreak of plant diseases or critical association of human pathogens with plants. We have learned that the human microbiome is much more involved in diseases than recently thought, and that pathogen outbreaks are associated with shifts in the entire community, including supporting pathogens (Blaser et al., 2013). While these processes are studied for human pathogens, much less is known about plant pathogens (Fürnkranz et al., 2012; Ottesen et al., 2013).

Furthermore, we envision the plant microbiome as an important source shaping other microbiomes. By the comparison of microbiome structures, a meaningful overlap of phylogenetic diversity can be recognized among microbiomes which are in some way linked to each other. This may also include the human habitat and plants. After we have received our first microbial inoculants by delivery and breast milk from our mother, our food becomes an important source not only of nutrients, but also of microorganisms (Blaser et al., 2013). Thus, digestive factors of plants and their microorganisms may modulate our own “second genome.” Observations of domestic microbiomes suggest that they are significantly influenced by their human inhabitants and by the surrounding vegetation (Oberauner et al., 2013). These connections, which we conceive as links in a complex network among microbiomes, are still little understood and need further attention.

## WHAT IS THE CONTRIBUTION OF THIS RESEARCH TOPIC?

This special issue will close some of the information gaps in plant microbiome ecology. It includes studies about the microbial diversity of yet unknown plants. In medicinal plants, the production of bioactive plant metabolites leads to a highly pronounced specificity in the microbiome structure (rev. in Köberl et al., 2014). Interestingly a correlation between the bioactive substances (drimane sesquiterpenes) and the endophytic community of roots was shown for the medical tree *Warburgia ugandensis* (Drage et al., 2014). Although it is known that plant secondary metabolites play an important role as drivers for microbial community structure, these studies show for the first time the importance with medicinal plants. Vice versa – Schmidt et al. (2014) could show that *Chamomile* plants treated with selected *Bacillus* strains produced more bioactive substances than untreated controls, thus microbes might be able to induce production of secondary metabolites of interest.

To better understand the significance of the plant-associated microbiome in prevention of pathogen outbreaks several studies focused on the lettuce microbiome and connected aspects of plant– and human health (Erlacher et al., 2014; Schreiter et al., 2014). Erlacher et al. (2014) showed that pathogens as well as beneficals induce a shift in the structure of the microbial community. To our knowledge, this is the first study analyzing this background effect, which can be important for plant protection strategies. However, also soil type was identified as important driver of the lettuce-associated community as well as the corresponding biocontrol effect (Schreiter et al., 2014). In addition, also for lettuce plants the impact of plant secondary metabolites exudated by roots in different soil types was pointed out (Neumann et al., 2014).

Another contribution presents evidence that *Escherichia coli* and *Salmonella enterica* infections occur due to consumption of vegetables, sprouts, and occasionally fruits (van Overbeek et al., 2014). The authors described a new transmission route of pathogens via plants or products derived from plants, and defined this process as “phytonosis”.

The role of multitrophic interactions for plant diseases and the occurrence of the western corn rootworm were analyzed by Dematheis et al. (2014). In addition to biotic factors, the impact of abiotic factors on the plant microbiome was investigated. Elevated atmospheric O_3_ changed the community structure of biocontrol active actinobacteria in the rhizosphere of European beech (Haesler et al., 2014).

Two studies suggest members of the plant-associated *Burkholderia* cluster as model to study plant-microbe interactions. Oxalate acts as carbon source and as determinant in colonization processes in lupins and maize (Kost et al., 2014), while nitrogen-fixing *Burkholderia* populations are highly abundant in *Sphagnum* bogs (Bragina et al., 2014).

Two mini-reviews focus on the interplay of microbiomes as well as the importance of the plant microbiome for others. The connection between plant and our built environment microbiome is discussed by Berg et al. (2014), and another one highlighted similarities between the gut and root microbiome and suggested to transplant “healthy microbiomes” to avoid or therapy plant diseases (Gopal et al., 2013). A step forward to understand the plant-microbe networking was presented in the review by Hartmann et al. (2014). They come to the conclusion that functional interaction studies of holobiotic plant systems, including the plant host and its associated microbes, may result in a more profound understanding of the complicated social network of basic innate immune responses with specific effector molecules, if quorum sensing compounds of endophytic bacteria are integrated.

Overall, this issue presents new results about (i) the role of plant secondary metabolites for the microbiome and *vice versa*, (ii) health issues related to the consumption of raw-eaten plants, (iii) the interplay of microbiomes as well as within them and (iv) the impact of biotic and abiotic factors on the structure and function of plant-associated microbial communities.

## Conflict of Interest Statement

The authors declare that the research was conducted in the absence of any commercial or financial relationships that could be construed as a potential conflict of interest.
